# Receptor-ligand supplementation via a self-cleaving 2A peptide–based gene therapy promotes CNS axonal transport with functional recovery

**DOI:** 10.1126/sciadv.abd2590

**Published:** 2021-03-31

**Authors:** Tasneem Z. Khatib, Andrew Osborne, Sujeong Yang, Zara Ali, Wanyi Jia, Ilya Manyakin, Katie Hall, Robert Watt, Peter S. Widdowson, Keith R. Martin

**Affiliations:** 1 Department of Clinical Neurosciences, University of Cambridge, Cambridge, UK.; 2 Eye Department, Cambridge University Hospitals NHS Foundation Trust, Cambridge, UK.; 3 Medical Sciences Division, University of Oxford, Oxford, UK.; 4 Quethera Ltd., Cambridge, UK.; 5 Ikarovec Ltd., Norwich Innovation Centre, Norwich, UK.; 6 School of Clinical Medicine, University of Cambridge, Cambridge, UK.; 7 Department of Physics, University of Cambridge, Cambridge, UK.; 8 Cambridge NIHR Biomedical Research Centre, Cambridge, UK.; 9 Wellcome Trust—MRC Cambridge Stem Cell Institute, University of Cambridge, Cambridge, UK.; 10 Ophthalmology, Department of Surgery, University of Melbourne, Melbourne, Australia.; 11 Centre for Eye Research Australia, Royal Victorian Eye and Ear Hospital, Melbourne, Australia.

## Abstract

Gene replacement approaches are leading to a revolution in the treatment of previously debilitating monogenic neurological conditions. However, the application of gene therapy to complex polygenic conditions has been limited. Down-regulation or dysfunction of receptor expression in the disease state or in the presence of excess ligand has been shown to compromise therapeutic efficacy. Here, we offer evidence that combined overexpression of both brain-derived neurotrophic factor and its receptor, tropomyosin receptor kinase B, is more effective in stimulating axonal transport than either receptor administration or ligand administration alone. We also show efficacy in experimental glaucoma and humanized tauopathy models. Simultaneous administration of a ligand and its receptor by a single gene therapy vector overcomes several problems relating to ligand deficiency and receptor down-regulation that may be relevant to multiple neurodegenerative diseases. This approach shows promise as a strategy to target intrinsic mechanisms to improve neuronal function and facilitate repair.

## Teaser

From vision impairment to memory loss, neurotrophin and receptor supplementation using gene therapy promotes functional recovery.

## INTRODUCTION

Gene replacement approaches to treat monogenic disease are leading to a revolution in the treatment of previously debilitating neurological conditions such as Leber’s congenital amaurosis (*1*), spinal muscular atrophy (*2*), and Leber’s hereditary optic neuropathy (LHON) (*3*). However, the application of gene therapy to complex polygenic conditions, which make up most of the neurodegenerative diseases, has been limited to date. Disruption of axonal transport is a hallmark of neurodegenerative disease, and stimulating axonal transport with the enhancement of intrinsic neuronal growth mechanisms in the diseased central nervous system (CNS) has been proposed as a promising strategy to improve neuronal repair.

Tropomyosin receptor kinase B (TrkB) promotion of axon growth has previously been described in regenerating corticospinal tract into subcortical lesions expressing brain-derived neurotrophic factor (BDNF) mediated through the extracellular signal–regulated kinase (ERK) signaling pathway (*4*) and has been shown to be crucial for neuronal migration, neurite outgrowth, differentiation, and connectivity (*5*, *6*). BDNF has also been implicated in axonal transport (*7*) and a self-amplifying positive feedback mechanism mediated through cyclic adenosine 5′-monophosphate (cAMP), protein kinase A, and the phosphatidylinositol 3-kinase (PI3 kinase) pathways to trigger further BDNF secretion, promote TrkB membrane insertion, and stimulate anterograde axonal transport, thereby contributing to axonal development and the formation of neuronal polarity (*8*). BDNF has also been shown to enhance TrkB receptor membrane stability and function by the recruitment of the receptor into lipid rafts of neuronal plasma membranes (*9*). However, the down-regulation or dysfunction of TrkB expression in the disease state (*10*) or in the presence of excess BDNF (*11*) has been shown to compromise therapeutic efficacy. We therefore hypothesized that combined BDNF and TrkB replacement would demonstrate superior efficacy compared to receptor or ligand alone and assessed this in two models of neurodegenerative disease known to be associated with reduced axonal transport (*12*–*14*).

Combination gene therapy, using multiple vectors administered in a single formulation, has been effective in targeting a diverse range of age-related pathologies in nonneuronal disease (*15*). However, dual promoter techniques have previously shown selective expression (*16*) and reduced efficiency (*17*). The simultaneous administration of a ligand and its receptor by a single self-cleaving 2A peptide–based gene therapy vector under the control of one promoter aims to overcome this by ensuring relatively long-term expression of both proteins in target cells (*18*). We assess this efficacy both in the presence of healthy endogenous receptors and ligand in vivo and in disease. We suggest that this approach overcomes several problems relating to ligand deficiency and receptor down-regulation in the diseased state or in the presence of excess ligand delivered therapeutically that may be relevant to multiple neurodegenerative diseases.

## RESULTS

### 2A linker region does not interfere with TrkB receptor activation segment

**Fig. 1:**
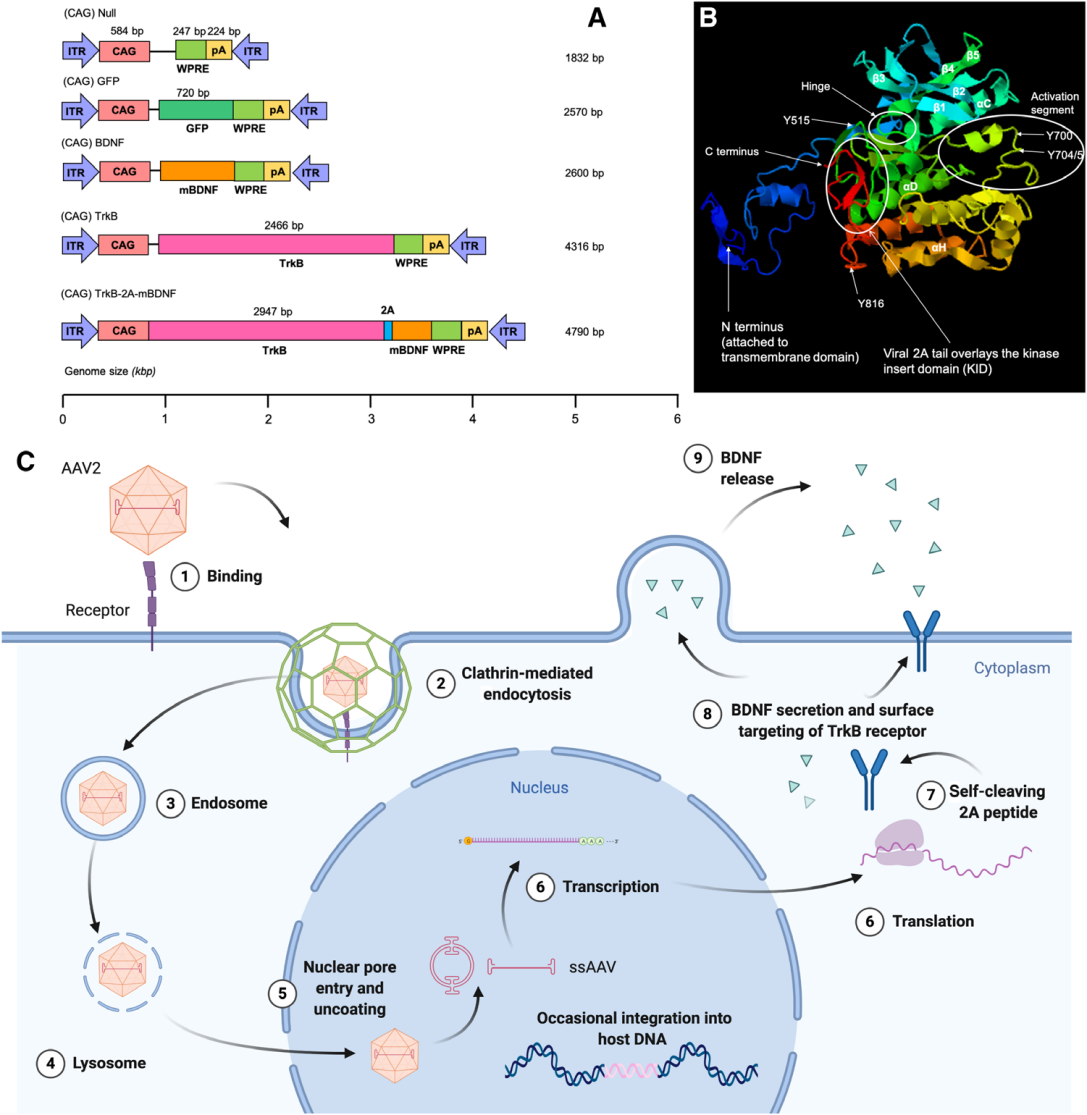
Simultaneous administration of a ligand and its receptor by a single self-cleaving 2A peptide–based gene therapy vector under the control of one promoter. (**A**) Plasmid maps (murine TrkB receptor). bp, base pair. (**B**) I-TASSER generated structure of TrkB receptor with residual 2A linker region. (**C**) Schematic demonstrating expression of TrkB and mBDNF in target cell.

Iterative Threading ASSEmbly Refinement (I-TASSER) was used to model the TrkB receptor administered via a single self-cleaving 2A peptide–based gene therapy vector under the control of one promoter (Fig. 1A) including the key tyrosine residues that are phosphorylated upon activation and the position of the 2A linker (Fig. 1B). The coding region for the TrkB receptor is linked to the coding region for mature BDNF (mBDNF) via the viral 2A peptide sequence. Intracellular processing of the transgene results in cutting of the viral 2A linker at the C-terminal proline-glycine and proline amino acids to generate the two proteins TrkB and mBDNF (Fig. 1C). While the N-terminal proline residue of the viral 2A peptide that is attached to the N terminus of the BDNF signal peptide will eventually be lost in the intracellular vesicles, before cellular release, 20 amino acids of the viral 2A peptide remain attached to the C-terminal TrkB intracellular kinase portion. The β-sheet layers (β1, β2, β3, β4, and β5) overlay the activation segment, which is circled in white. The active site contains the central tyrosine residues (Y700, Y704, and Y705), which are phosphorylated upon dimerization in the presence of BDNF. The viral 2A peptide C-terminal extension (Fig. 1B, red) is shown to overlay the kinase insert domain, which lies at the foot of the helix complex (αD). The viral 2A peptide extension does not lie close to the activation segment or interfere with the hinge region necessary for receptor conformational changes following phosphorylation and subsequent activation.

### Transduction efficiency of the rodent retina

**Fig. 2:**
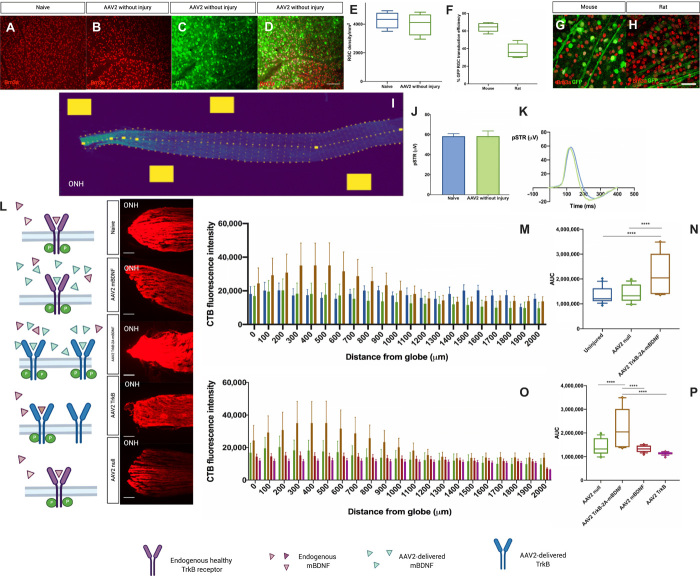
Combined ligand receptor replacement more effective than ligand or receptor alone. (**A** to **E**) AAV2 injection not associated with RGC loss [naive 4279 ± 294 RGCs/mm^2^ compared to AAV2 injection without injury 4004 ± 391 RGCs/mm^2^, *P* = 0.59 (*n* = 4)]. (**F** to **H**) Transduction of Brn3a-positive RGCs in mouse and rat [mouse transduction efficiency, 64.01 ± 2.02%; rat transduction efficiency, 37.61 ± 3.36% (*n* = 6)]. (**I**) Semiautomated quantification of anterograde optic nerve axonal transport. Yellow boxes represent automated background fluorescence measurements taken and used to normalize readings. (**J** and **K**) AAV2 injection does not impair RGC function [naive 58.38 ± 2.52 μV compared to AAV2 injection without injury 58.41 ± 5.20 μV, *P* = 0.99 (*n* = 9)]. (**L**) Schematic of AAV2 therapy with corresponding representative images of the proximal optic nerve with CTB. (**M** and **N**) AAV2 TrkB-2A-mBDNF enhances anterograde axonal transport in the uninjured optic nerve [uninjured, 1,356,991 ± 74,285 (*n* = 13); AAV2 null, 1,407,781 ± 76,284 (*n* = 4); AAV2 TrkB-2A-mBDNF, 2,224,777 ± 186,617 (*n* = 5)]. AUC, area under the curve. (**O** and **P**) Combined ligand receptor replacement is more effective than ligand or receptor alone [AAV2 TrkB-2A-mBDNF, 2,151,629 ± 191,990 (*n* = 5); AAV2 TrkB, 1,138,337 ± 13,648 (*n* = 4); AAV2 mBDNF, 1,327,883 ± 30,372 (*n* = 5)]. Data are means ± SEM. Box and whiskers plots: Median displayed and whiskers 10th to 90th percentile with any outliers displayed as individual data points. Scale bar, 100 μm. ****P < 0.0001, Student’s *t* test and ANOVA with Bonferroni correction.

Intravitreal injection of the vector did not adversely affect retinal ganglion cell (RGC) density (Fig. 2, A to E, and fig. S1A). The vector transduced both mouse and rat retinal RGCs (Fig. 2, F to H, and fig. S2A) in addition to other retinal cell types including bipolar, horizontal, Muller, and amacrine cells (fig. S2, B to E). There was a significant increase in expression of BDNF and TrkB following retinal transduction with adeno-associated virus 2 (AAV2) TrkB-2A-mBDNF relative to AAV2 null (fig. S3).

### Combined replacement is more effective than receptor or ligand replacement alone

Proximal optic nerve axonal transport (measured as shown in Fig. 2I) increased following administration of AAV2-TrkB-2A-mBDNF in the uninjured optic nerve compared with naive levels (Fig. 2, L to N) along the length of the nerve. The greatest effect was observed in the proximal 1 mm closest to the optic nerve head, where the site of obstruction to axoplasmic flow has previously been reported to occur in glaucoma (*13*, *14*). This enhancement of anterograde axonal transport was independent of any neuroprotective effect (Fig. 2, A to E), as there was no change in the density of Brn3a-positive RGC bodies following intravitreal gene therapy administration. AAV2 injection did not affect RGC function as measured by the positive scotopic threshold response (pSTR) (Fig. 2, J and K). The effect of combined TrkB and mBDNF administration in the single vector on axonal transport was superior to that seen with either AAV2 TrkB or AAV2 mBDNF alone (Fig. 2, O and P) in the presence of healthy endogenous receptor or ligand afforded by the absence of injury.

### Intervention is effective after the onset of pathology in a humanized tauopathy model

**Fig. 3:**
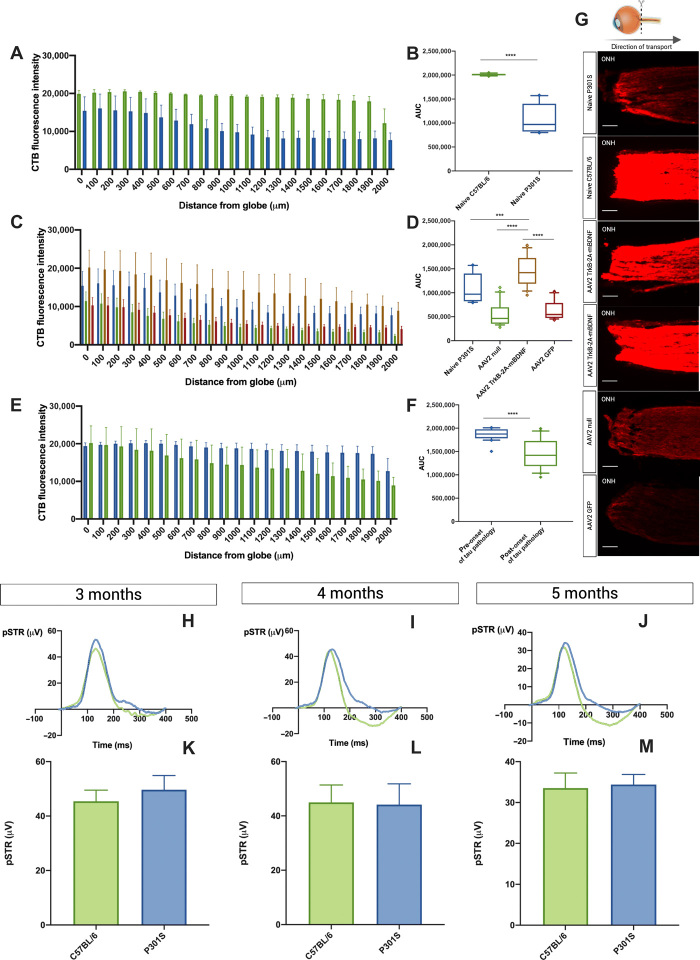
Intervention is effective after the onset of pathology in a humanized tauopathy model. (**A** and **B**) Reduction in anterograde optic nerve axonal transport of CTB in P301S transgenic mice [C57BL/6 2,009,762 ± 4629 (*n* = 3) compared to 5-month P301S 1,085,928 ± 67,416 (*n* = 8)]. (**C** and **D**) AAV2 TrkB-2A-mBDNF enhances optic nerve axonal transport after the onset of tau pathology [AAV2 TrkB-2A-mBDNF, 1,454,297 ± 70,125 (*n* = 7); naive, 1,085,928 ± 67,416 (*n* = 8); AAV2 null, 552,603 ± 54,452 (*n* = 4); AAV2 GFP, 631,690 ± 44,433 (*n* = 5)]. (**E** and **F**) AAV2 TrkB-2A-mBDNF is more effective if administered before the onset of tau pathology [intervention before onset, 1,863,778 ± 27,856 (*n* = 5); after onset, 1,454,297 ± 70,125 (*n* = 7)]. (**G**) Direction of axon transport and representative images of the proximal optic nerve with CTB. Third panel down from top, AAV2 TrkB-2A-mBDNF administered after onset of tau pathology; fourth panel down from top, AAV2 TrkB-2A-mBDNF administered before onset of tau pathology. (**H** to **M**) Anterograde optic nerve axonal transport deficits precede RGC functional deficits in P301S transgenic mice pSTR in 3-month (H and K), 4-month (I and L), and 5-month (J and M) P301S transgenic mice [(H) and (K): 3-month C57BL/6 45.4 ± 4.10 μV (*n* = 16) compared to P301S 49.7 ± 5.25 μV (*n* = 9); (I) and (L): 4-month C57BL/6 45.0 ± 6.40 μV (*n* = 10) compared to P301S 44.1 ± 7.66 μV (*n* = 8); (J) and (M): 5-month C57BL/6 33.5 ± 3.69 μV (*n* = 13) compared to P301S 34.3 ± 2.47 μV (*n* = 12)]. Data are means ± SEM. Box and whiskers plots: Median displayed and whiskers 10th to 90th percentile with any outliers displayed as individual data points. Scale bar, 100 μm. ***P < 0.001 and ****P < 0.0001, Student’s *t* test and ANOVA with Bonferroni correction.

We explored whether this effect could be applied in the disease state, using the P301S humanized tauopathy model. Filamentous inclusions made of hyperphosphorylated tau are present in the CNS of 3- and 5-month-old transgenic mice including RGCs (*19*). Figure 3 (A and B) shows a reduction in anterograde axonal transport in the optic nerves of 5-month-old P301S mice compared to age-matched C57BL/6 mice. This reduction in transport was not associated with any reduction in RGC count (fig. S4, A to C).

There was a significant increase in proximal optic nerve axonal transport with AAV2 TrkB-2A-mBDNF in the tauopathy model administered after the onset of tau pathology relative to naive, AAV2 null, and AAV2 green fluorescent protein (GFP) controls (Fig. 3, C and D). Administering the vector before tau pathology onset had greater efficacy than when administered in the pathological state (Fig. 3, E and F). Representative images of cholera toxin B protein subunit b (CTB) in the proximal optic nerve are shown in Fig. 3G. Disruption in axonal transport occurred before observable functional loss, representing a possible early therapeutic target. There was no reduction in RGC function in the P301S tauopathy model up to the 5-month time point (Fig. 3, H to M).

### AAV2 TrkB-2A-mBDNF enhances axonal transport in experimental glaucoma with functional recovery

**Fig. 4:**
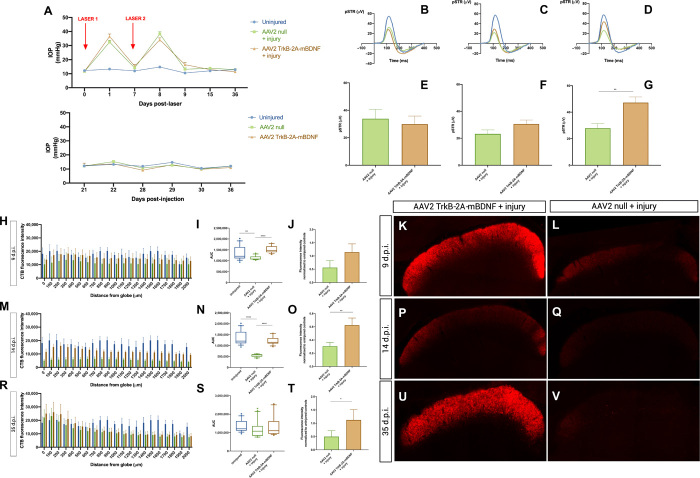
AAV2 TrkB-2A-mBDNF enhances anterograde optic nerve axonal transport in experimental glaucoma with functional recovery. (**A**) Top: Baseline IOP (uninjured, 12.2 ± 0.29 mmHg; AAV2 null, 11.5 ± 0.28 mmHg; AAV2 TrkB-2A-mBDNF, 11.6 ± 0.24 mmHg). IOP elevation [AAV2 null (D1, 32.4 ± 1.35 mmHg; D7, 14.1 ± 0.76 mmHg; D8, 38.2 ± 1.56 mmHg; D9, 13.2 ± 1.06 mmHg; D15, 13.9 ± 0.80 mmHg; D36, 12.9 ± 0.70 mmHg) and AAV2 TrkB-2A-mBDNF (D1, 36.5 ± 1.73 mmHg; D7, 15.4 ± 1.19 mmHg; D8, 34.0 ± 1.44 mmHg; D9, 16.4 ± 1.62 mmHg; D15, 13.0 ± 0.0 mmHg; D36, 11.4 ± 0.45 mmHg)]. Bottom: AAV2 TrkB-2A-mBDNF does not affect IOP. (**B** to **G**) Functional deficits precede transport deficits and are restored with AAV2 TrkB-2A-mBDNF. pSTR D9 [(B) and (E): AAV2 null + injury, 33.8 ± 6.81 μV; AAV2 TrkB-2A-mBDNF + injury, 29.9 ± 5.94 μV (*n* = 9)], D14 [(C) and (F): AAV2 null + injury, 23.2 ± 2.99 μV (*n* = 24); AAV2 TrkB-2A-mBDNF + injury, 30.4 ± 3.00 μV (*n* = 21)], and D35 [(D) and (G): AAV2 null + injury, 27.9 ± 3.32 μV (*n* = 23); AAV2 TrkB-2A-mBDNF + injury, 47.2 ± 4.34 μV (*n* = 20); *P* = 0.002]. (**H**, **I**, **M**, **N**, **R**, and **S**) Optic nerve axonal transport D9 [(H) and (I): uninjured, 1,356,991 ± 74,285 (*n* = 13); AAV2 null + injury, 1,123,294 ± 21,687 (*n* = 4); AAV2 TrkB-2A-mBDNF + injury, 1,517,967 ± 35,554 (*n* = 5)], D14 [(M) and (N): uninjured, 1,356,991 ± 74,285 (*n* = 13); AAV2 null + injury, 549,638 ± 15,124 (*n* = 4); AAV2 TrkB-2A-mBDNF + injury, 1,214,833 ± 40,492 (*n* = 5); *P* < 0.0001], and D35 [(R) and (S): AAV2 null + injury, 1,192,000 ± 102,085 (*n* = 8); AAV2 TrkB-2A-mBDNF + injury, 1,349,326 ± 124,332 (*n* = 10)]. (**J** to **L**, **O** to **Q**, and **T** to **V**) Anterograde transport to the superior colliculus D9 [(J) to (L): AAV2 null + injury, 0.56 ± 0.26–fold reduction from uninjured (*n* = 4); AAV2 TrkB-2A-mBDNF + injury, 1.14 ± 0.31–fold increase from uninjured (*n* = 5); *P* = 0.39], D14 [(O) to (Q): AAV2 null + injury, 0.30 ± 0.11–fold reduction from uninjured (*n* = 4); AAV2 TrkB-2A-mBDNF + injury, 0.62 ± 0.11–fold reduction from uninjured (*n* = 5); *P* = 0.009], and D35 [(T) to (V): AAV2 null + injury, 0.50 ± 0.22–fold reduction from uninjured (*n* = 9); AAV2 TrkB-2A-mBDNF + injury, 1.12 ± 0.39–fold increase from uninjured (*n* = 7); *P* = 0.02]. Data are means ± SEM. Box and whiskers plots: Median displayed and whiskers 10th to 90th percentile with outliers displayed as individual data points. *P < 0.05, **P < 0.01, ***P < 0.001, and ****P < 0.0001, Student’s *t* test and ANOVA with Bonferroni correction. d.p.i., days post-injury.

Figure 4 shows anterograde optic nerve axonal transport in our experimental glaucoma model, which is known to cause RGC degeneration and disruption of axonal transport due to intraocular pressure (IOP) elevation (*13*, *14*, *20*–*22*). Intravitreal injection of AAV2-TrkB-2A-mBDNF or with AAV2 null alone did not produce any changes in the IOP, as measured immediately before laser treatment (Fig. 4A, top) or during the course of the experiment when injected into the nonlasered eye (Fig. 4A, bottom). IOP elevation was similar for both AAV2 null and AAV2 TrkB-2A-mBDNF groups across all time points and returned to baseline levels 1 week following the second laser insult, remaining at baseline levels for the duration of the study (Fig. 4A).

There was a reduction in the RGC-specific pSTR in both groups at 2 days (Fig. 4, B and E) and 1 week (Fig. 4, C and F) following experimental IOP elevation, with functional recovery at 1 month in the AAV2 TrkB-2A-mBDNF–treated group (Fig. 4, D and G). Proximal optic nerve axonal transport decreased early after injury at 2 days (Fig. 4, H and I) and 1 week (Fig. 4, M and N) in the AAV2 null–treated group and remained at baseline levels in the AAV2 TrkB-2A-mBDNF group. This reduction in transport and function was not associated with RGC loss (fig. S4). The proximal transport started to recover at 1 month after injury (Fig. 4, R and S), but distal transport at the level of the superior colliculus remained depressed in the AAV2 null–treated group (Fig. 4, T to V). 

### Translating from the visual pathway to memory: AAV5 TrkB-2A-mBDNF improves short-term memory loss in a humanized dementia model of Alzheimer’s disease

**Fig. 5:**
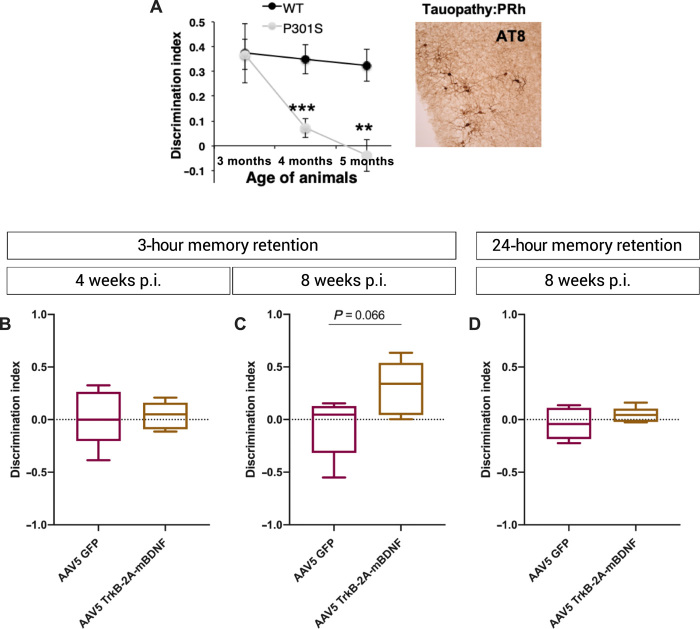
AAV5 TrkB-2A-mBDNF improves short-term memory in a humanized tauopathy model of dementia. (**A**) Memory deficit in the P301S tauopathy model. Reprinted with permission from Yang *et al*. (*65*). (**B**) Three-hour memory retention at 4 weeks after injection [AAV5 GFP 0.02 ± 0.12 discrimination index compared with AAV5 TrkB-2A-mBDNF 0.04 ± 0.06 discrimination index; *P* = 0.92 (*n* = 5)]. (**C**) AAV5 GFP 0.07 ± 0.13 discrimination index compared with AAV5 TrkB-2A-mBDNF 0.30 ± 0.16 discrimination index; *P* = 0.066 (*n* = 5). (**D**) AAV5 GFP 0.03 ± 0.07 discrimination index compared with AAV5 TrkB-2A-mBDNF 0.04 ± 0.03 discrimination index; *P* = 0.32 (*n* = 5). Data are as means ± SEM. Box and whiskers plots: Median displayed and whiskers 10th to 90th percentile with any outliers displayed as individual data points. Student’s *t* test used as statistical test.

The eye is widely considered to be an accessible experimental model of the CNS, and there have been recent correlations in amyloid-related pathology in the brain and retina in Alzheimer’s disease (*23*, *24*). We therefore explored whether the improvement in optic nerve axonal transport seen in the P301S model using AAV2 TrkB-2A-mBDNF could be extrapolated to the brain and specifically memory using the spontaneous object recognition test. We used the same P301S humanized transgenic tauopathy line used in the optic nerve experiments as our dementia model (Fig. 5, A and B). Injection of AAV5 TrkB-2A-mBDNF into the perirhinal cortex was associated with promising evidence that there may be an improvement in short-term memory at 8 weeks after injection (Fig. 5C). Longer-term memory deficit, however, persisted at the 8-week time point (Fig. 5D).

## DISCUSSION

Here, we offer evidence that the combined overexpression of both BDNF and its receptor TrkB, by a single vector with one promoter, is more effective in stimulating anterograde axonal transport than either receptor administration or ligand administration alone. The position of the viral 2A peptide extension on the TrkB receptor does not lie close to the activation segment, and we have previously demonstrated that phosphorylation of the receptor is unaffected by the presence of the 2A linker with subsequent up-regulation of downstream survival signaling pathways (*18*, *25*).

We also report that simultaneous overexpression of BDNF and TrkB enhances axonal transport in two neurodegenerative disease models: a humanized tauopathy model of Alzheimer’s disease and an experimental glaucoma model. We also show that deficits in axonal transport occur early in the tauopathy model before functional changes, representing a possible early therapeutic target. Functional recovery is also demonstrated in our experimental glaucoma model, and we show that there may be an improvement in short-term memory deficit in a humanized dementia model.

This improvement in axonal transport is independent of any BDNF-mediated neuroprotective increase in cell body or axon number, effects that have been previously reported (*18*, *26*), as it occurs without RGC loss in naive animals, early after injury and in the P301S tauopathy model of reduced axonal transport. Intervention is effective in our disease models and remains effective if administered after the onset of pathology, suggesting a clinically relevant therapeutic window.

The underlying mechanisms through which the BDNF/TrkB pathway promotes axonal transport remain poorly understood. TrkB promotion of axon growth has been reported in regenerating corticospinal tract into subcortical lesions expressing BDNF mediated through the ERK signaling pathway (*4*). BDNF has also been implicated in a self-amplifying positive feedback mechanism mediated through cyclic AMP, protein kinase A, and the PI3 kinase pathways to trigger further BDNF secretion, promote TrkB membrane insertion, and stimulate anterograde axonal transport (*8*). BDNF has also been shown to enhance TrkB receptor membrane stability and function by the recruitment of the receptor into lipid rafts of neuronal plasma membranes (*9*). Elevated levels of BDNF with TrkB activation of the PI3 kinase pathway and subsequent inactivation of glycogen synthase kinase-3β also modulate tau pathology and hyperphosphorylation (*27*) and may reduce the progression of tau pathology with associated microtubule instability and axonal transport disruption (*10*, *12*, *28*). Similar effects have been suggested following exercise-induced elevation of BDNF (*29*, *30*) and exercise-mediated reduction in CNS tau pathology in P301S mice (*31*). Abnormalities in tau phosphorylation have also been implicated in the glaucomatous degenerative disease process in patients with intractable glaucoma not responsive to conventional therapies (*32*). However, exogenous BDNF in the absence of TrkB overexpression has not been sufficient to promote axon outgrowth in the diseased state in P301S retinal explants (*19*). The demonstration of enhanced axonal transport above naive levels in adult wild-type Sprague Dawley rats lends support to previous findings that there may be a decline in axonal transport mechanisms with age including young adulthood (*33*) independent of injury and that mature neurons retain the capacity for enhancement and ability to support a higher transport rate. An evaluation of the impact of the therapy on retrograde axonal transport, which has also been implicated in glaucomatous pathology, together with a dynamic assessment of the transport of endogenous proteins would address some of the limitations of this study.

The relationship between axonal transport and cellular function is complex, and as we have demonstrated here, the disruption of function can occur both early and later in the disease process. We have demonstrated functional recovery where functional deficits occurred before axonal transport loss. Impaired inner retinal function has been shown to precede defects in anterograde active axonal transport of CTB following IOP elevation (*34*, *35*), with a redistribution in the size and location of the axon initial segment postulated to alter action potential propagation following glaucomatous injury. Neuronal excitability has been reported as a response to IOP elevation (*36*), and visual stimulation and electrical activity have also been shown to influence axonal regeneration (*37*).

We observed recovery of the pSTR in our AAV2 TrkB-2A-mBDNF–treated group following IOP elevation. This corresponds with functional recovery following IOP elevation previously described (*38*–*40*) and supports the possibility of reversal of glaucomatous dysfunction of RGCs and their connections, lending further support to the use of this treatment approach in the development of a clinical therapy for glaucoma. The construct is likely to have additional effects on neuronal connectivity and activity, and our data would suggest that the effects observed cannot be attributed to transport alone. BDNF has also been shown to prevent dendritic retraction of RGCs (*41*) and enhance synaptic activity (*42*, *43*), and autocrine BDNF/TrkB signaling on dendritic spines has been implicated in modulating structural and functional plasticity (*44*). The observation that the CTB intensity was reduced in the distal optic nerve but maintained in the superior colliculus following administration of AAV2 TrkB-2A-mBDNF in the experimental glaucoma model could be explained by improved neuronal connectivity or regeneration at the level of the colliculus. Transduction of nontarget cells may also contribute, and glial BDNF (*11*) and TrkB (*45*, *46*) signaling has previously been shown to play an important role in neural protection after traumatic optic nerve injury and can delay retinal degeneration. The improvement in short-term memory in the humanized tauopathy dementia model is consistent with previous studies (*47*–*49*) and lends further support to the use of the eye and visual pathway as an accessible system to investigate CNS neurodegeneration. Whether the timing and pattern of delivery to the cortex can be optimized to affect longer-term memory loss remains of interest. Structural comparison between the effect of BDNF and TrkB overexpression on cortical and retinal pathology should also be a focus of future work. It is therefore conceivable that overexpression of BDNF and TrkB could act through multiple mechanisms, by restoring neurotrophin support that has been lost through a disruption of axonal transport (*50*), boosting the ability of target cells to withstand pathological insults, and manipulating the underlying pathological processes that contribute to disruption of neuronal integrity and function.

We have previously discussed limitations associated with translation from preclinical to clinical studies for non-IOP lowering novel glaucoma therapies (*51*). Refinements in clinical trial design together with optimization of structural and functional endpoints are crucial in the assessment of future therapies. Allometric scaling and the pharmacokinetics of the human vitreous and the internal limiting membrane have also been suggested as potential barrier to viral transduction of the retina following intravitreal injection. We have demonstrated efficacy despite a reduction in transduction efficiency in the rat RGCs compared to mouse RGCs. It is of interest that the rAAV2/2-ND4 clinical trial for LHON demonstrated efficacy following intravitreal injection in a younger LHON cohort who typically has a thicker internal limiting membrane and denser vitreous composition (*52*). It remains to be seen whether the presence of vitreous syneresis or posterior vitreous detachment in the more elderly glaucoma patient population will lessen the structural barrier to viral transduction, hence improving transgene expression. The AAV8-RS1 gene therapy trial for X-linked retinoschisis also uses an intravitreal approach to administer a healthy copy of retinoschisis to target photoreceptors. Cukras *et al.* (*53*) postulated that the retinal degeneration associated with the X-linked retinoschisis also compromises the structural integrity of the internal limiting membrane, facilitating retinal penetration following intravitreal administration of the viral vector. Our intervention was administered before the induction of disease in the experimental glaucoma model. We feel that these findings do have implication for clinical practice. The increasing role of artificial intelligence and functional imaging techniques have the potential to significantly improve our early detection of at-risk patients before the appearance of symptoms, together with progress in the molecular profiling of glaucoma patients and polygenic risk score stratification of disease progression. We have also shown that early intervention is more effective than late intervention in the tauopathy model of reduced axonal transport, which also lends further support toward the role of early intervention.

In summary, we have demonstrated an enhancement of axonal transport in RGC axons in two disease models using AAV2 TrkB-2A-mBDNF and suggest that enhancements in the visual pathway can be translated to other regions in the CNS. We also show promising evidence that there may be an improvement in short-term memory deficit using AAV5 TrkB-2A-mBDNF in a humanized tauopathy model of dementia. Given the recent correlation between Alzheimer’s pathology in the brain and retina (*23*, *24*), this approach shows promise as an overall strategy to target intrinsic neuronal mechanisms to improve neuronal repair while overcoming several problems relating to ligand deficiency and receptor down-regulation and dysfunction that may be relevant to multiple neurodegenerative diseases.

## MATERIALS AND METHODS

### Vector production

AAV vectors were manufactured by Vigene Biosciences (Rockville, MD, USA). Vector particles were liberated following freeze-thaw of human embryonic kidney (HEK) 293 cells transduced with plasmid DNA, followed by iodixanol gradient ultracentrifugation and desalting, and suspended in phosphate-buffered saline (PBS) buffer (Thermo Fisher Scientific). Titers were confirmed by Vigene Biosciences through quantitative polymerase chain reaction using primers recognizing the inverted terminal repeat regions. All vectors contained a modified CAG promoter, shortened Woodchuck hepatitis virus posttranscriptional regulatory element, and simian virus 40 late polyadenylation signal, as described previously (*25*).

### Animals

Homozygous mice transgenic for human mutant P301S tau [original colony from M. Goedert (*54*)] were used at 1, 3, and 4 months of age. These mice are bred on a C57BL/6 background, and the P301S mutation is under the control of the Thy-1 promoter. Filamentous inclusions made of hyperphosphorylated tau are present in the CNS of 3- and 5-month-old transgenic mice, and we have previously described the phenotypic appearance of the retina and optic nerve, expression of human mutant P301S tau in RGCs, and the formation of tau inclusions (*19*) together with a reduction in optic nerve axonal transport and an increased susceptibility to injury (*12*). Age- and sex-matched C57BL/6 mice (Charles River Laboratories, UK) were used as controls. Wild-type adult Sprague-Dawley rats were used for the laser-induced ocular hypertension model of experimental glaucoma. Animals had unrestricted access to food and water and were maintained on a 12-hour light/dark cycle. All experiments were carried out in accordance with the UK Home Office Regulations for the Care and Use of Laboratory Animals and the UK Animals (Scientific Procedures) Act 1986 and the Association for Research in Vision and Ophthalmology’s Statement for the Use of Animals in Ophthalmic and Visual Research. They were approved by the University of Cambridge Animal Ethics Committee (project license 70/8152).

### Intravitreal vector injection

Animals were anesthetized with intraperitoneal injection of ketamine (50 mg/kg) and xylazine (10 mg/kg) and topical 1% tetracaine eye drops (Bausch & Lomb) before injection. Freshly thawed AAV2 vectors were diluted to working stock in sterile PBS and 2 μl (mice) or 5 μl (rats) of vector injected through the sclera into the vitreous of the eye posterior to the limbus (syringe: 5 μl, #65RN; needle: ga33, 8mm, pst2, Hamilton Co.). Injections were given slowly over 1 min to allow diffusion of the vector suspension. All injections were carried out by the same surgeon, and vectors were administered at 1 × 10^10^ genomic copies per eye. Tissue was collected at the indicated time points following transcardial perfusion with 4% paraformaldehyde (PFA) under terminal anesthesia.

### Rat laser-induced ocular hypertension

Twenty-one days after intravitreal vector injection, ocular hypertension was induced in rats as previously described (*22*). Rats were placed in front of a slit-lamp, and 40 to 60 laser pulses (wavelength, 532 nm; spot size, 50 μm; power, 700 mW; duration, 600 ms) were directed around the circumference of the trabecular meshwork to impair aqueous drainage. Contralateral fellow eyes served as normotensive controls. IOP was measured bilaterally under anesthesia on procedure days and while awake at subsequent time points using a TonoLab rebound tonometer calibrated for the rat eye (Tiolat Oy). Tonometry was performed within 5 min of anesthesia onset and between the hours of 9:00 and 11:00 a.m. Fifteen recordings were taken from each eye at each time point.

### Electroretinography

Full-field electroretinographies (ERGs) were recorded simultaneously from both eyes of each animal as described previously (*55*). Animals were dark-adapted overnight, and ERG recordings were performed under low-level, red-light illumination. ERG recordings were acquired using an Espion E3 system with full-field Ganzfeld sphere (Diagnosys, Cambridge, UK) as follows: Scotopic threshold response recorded at −4.73 log cd s m^−2^ (averaged responses from between 20 recordings with interstimulus interval of 3 s). Peak amplitude between 80 and 120 ms was taken as the pSTR amplitude.

### Spontaneous object recognition task

The spontaneous object recognition task was performed as previously described (*56*, *57*), using a Y-shaped apparatus. Briefly, all mice were habituated in three consecutive daily sessions in the empty Y-maze apparatus for 5 min. Each test session consisted of a sample phase and a choice phase. In the sample phase, the animal was placed in the start arm and left to explore the two identical objects, which were placed on the end of two arms for 5 min. The choice phase followed after a delay of either 3 or 24 hours during which the animal spent in the home cage. The choice phase was procedurally identical to the sample phase, except that one arm contained a novel object, whereas the other arm contained a copy of the repeated object. An unused copy of the repeated object was used to avoid olfactory disturbance. Each animal received two test sessions for each delay. A different object pair was used for each session for a given animal, and the order of exposure to object pairs as well as the designated sample and novel objects for each pair were counterbalanced within and across groups. The object exploration time was assessed from video recordings of the sample and choice phase. Direct nasal or head contacts only were regarded as an exploratory behavior. For the choice phase, a discrimination ratio was calculated by dividing the difference in exploration of the novel and familiar objects by the total object exploration time. Therefore, the discrimination ratio varies from 0 (equal exploration for novel and familiar objects) to 1 (exploration of the novel object only). The mean discrimination ratio across two test sessions was calculated for each animal.

### Perirhinal cortex AAV5 injections

Six injections into the perirhinal cortex (three per hemisphere, 0.5 μl with a speed of 0.2 μl/min) were performed stereotaxically under isoflurane anesthesia with a 10-μl Hamilton syringe (syringe: 10 μl, #701RN; needle: ga33, Hamilton Co.) and a 33-gauge needle at the following sites (from bregma and the surface of skull): (i) anterior-posterior (AP): −1.8 mm, lateral (L): ±4.6 mm, and ventral (V): −4.4 mm; (ii) AP: −2.8 mm, L: ±4.8 mm, and V: −4.3 mm; and (iii) AP: −3.8 mm, L: ±4.8 mm, and V: −3.8 mm. The needle remained in place at the injection site for 3 min before being slowly withdrawn over 2 min.

### Western blots

Retinas were excised from the eye cup immediately after death, and tissue was snap-frozen on dry ice before lysing using a Lysis-M reagent containing cOmplete Mini Protease Inhibitor (Roche) and phosphatase inhibitors (Thermo Fisher Scientific). Following 20-min homogenization, tissue was centrifuged at 13,000 rpm for 10 min to isolate the soluble cell extract. Protein concentration was determined using a bicinchoninic acid protein assay (Thermo Fisher Scientific), and equal quantities of protein were loaded onto 10% or 4 to 12% bis-tris gels (NuPAGE Novex, Thermo Fisher Scientific). Membranes were blocked in 5% dried skimmed milk in PBS with 0.2% Tween 20 (Sigma-Aldrich) for 60 min and then incubated overnight at 4°C in primary antibody (BDNF N-20, rabbit, 1:200, sc-546, Santa Cruz Biotechnology; TrkB, rabbit, 1:500, ab33655, Abcam; β-actin, rabbit, 1:1000, 4967, Cell Signaling Technology). Primary antibodies were visualized with horseradish peroxidase–conjugated anti-rabbit secondary antibody (1:8000; PI-1000, Vector Laboratories) and signal detection using ECL Prime (GE Healthcare) and an Alliance Western blot imaging system (UVItec Ltd.).

### Measuring anterograde axonal transport

#### Tracing the optic nerve

The vitreous body of the left eye was injected with 2 μl of 0.1% solution of CTB conjugated to Alexa Fluor (AF) 555 in sterile PBS (Thermo Fisher Scientific). CTB binds the GM1 ganglioside receptor on RGCs and is widely used as an axonal tracer and marker of transport because it is selectively taken up by RGCs in the retina (*58*). Following the injection, the needle was held in place for 1 min to prevent reflux. Animals were perfused intracardially with 4% PFA 24 hours after CTB administration.

#### Tissue processing

Optic nerves of perfused mice were dissected immediately behind the posterior globe at the optic nerve head and the optic chiasm, and brains were removed. Nerve and brain tissue was postfixed overnight at 4°C, washed in PBS, and cryopreserved by overnight immersion in 30% sucrose at 4°C. Following embedding in optimal cutting temperature compound (Tissue-Tek, Miles Laboratories), serial 30-μm-thick coronal cryo-sections of the entire superior colliculus from each animal were obtained and 14-μm-thick longitudinal sections of the optic nerve were cut using a cryostat and mounted onto microscope slides in darkened conditions. Immunohistochemical visualization of CTB was not required, as the fluorophore was directly conjugated to the CTB. Sections were visualized under epifluorescent illumination on a single Leica DMi8 microscope using a 20× objective (optic nerve) and 10× objective (superior colliculus). Contiguous images were captured and tiled using LAS AF software (Leica Inc.) and identical camera settings.

#### Quantification of anterograde axonal transport of CTB

We developed a semiautomated tool (Fig. 2I) to quantify mean CTB fluorescence intensity across the width of each optic nerve, perpendicular to the long axis of the nerve, at 100-μm intervals as previously described (*12*). Images were segmented by applying a normalized box filter to remove high spatial frequency noise followed by binary thresholding. A bespoke graphic user interface permitted manual segmentation if required to optimize the analysis.

Intermediate waypoints with a user-defined spacing (100 μm here) along the proximal-distal longitudinal axis of the optic nerve were generated using

$$P_{n}=P_{0}+n\cdot d\cdot {\hat{v}}_{∥}$$

where *P*_0_ is the start waypoint, *n* is the integer index of the intermediate waypoint, *d* is a user-defined interwaypoint distance, and ${\hat{v}}_{∥}$ is the normalized unit vector parallel to the segment defined by the start and end waypoints. Pixel coordinates were produced along the line passing through the intermediate waypoint *P*_n_ that is perpendicular to the segment, and pixel intensity was normalized to three independent measures of background fluorescence. Intensity measures were plotted against distance along the nerve from the optic nerve head, and the area under the curve was calculated. Ninety-nine percent crossover of axons occurs at the optic chiasm in albino strains (*59*, *60*), and the mean corrected total CTB fluorescence from six to nine sections of the contralateral superior colliculus was taken to represent distal anterograde transport for vector-treated or control groups as previously described (*61*).

### Immunohistochemistry

#### Whole mounts

Retinal whole mounts were prepared by removal of the anterior segment, lens, and vitreous from the globe and dissection of the neurosensory retina from the underlying retinal pigment epithelium. Retinas were flattened and postfixed for 30 min in 4% PFA before immunohistochemistry. Whole mounts were washed in 0.5% Triton X-100 in PBS and frozen at −80°C for 10 min to permeate the nuclear membrane and improve antibody permeation before blocking in 10% normal donkey serum, 2% bovine serum albumin (BSA), and 2% Triton X-100 in PBS for 60 min. RGCs were labeled with Brn3a (C-20, goat, 1:200, sc-31984, Santa Cruz Biotechnology), TUJ1 (mouse, 1:500, G7121, Promega), and RBPMS (guinea pig, 1:500, 1832, PhosphoSolutions) diluted in blocking solution for 2-hour incubation at room temperature and then at 4°C overnight. Donkey anti-mouse AF488 (1:1000, a32766, Invitrogen), donkey anti–guinea pig AF555 (1:500, SAB4600297, Sigma-Aldrich), and donkey anti-goat AF555 or AF647 (1:500, a32816 or 1:500, a21447, Invitrogen) were used as secondary antibodies in 2% PBS–Triton X-100 with a 2-hour incubation period at room temperature followed by three wash steps in PBS. Retinal whole mounts were mounted onto slides with FluorSave reagent (345789, MilliporeSigma). Whole mounts were visualized under epifluorescent illumination on a Leica DMi8 microscope using a 20× objective. RGC counts were measured using Fiji (*62*) and an image-based tool for counting nuclei (ITCN) plugin as previously described (*18*). Volocity software was used to quantify transduction efficiency by determining the percentage colocation of GFP expressed in Brn3a-positive RGCs. Colocalization of the RGC markers used in this study (Brn3a, TUJ1, and RBPMS) is demonstrated in fig. S5.

#### Sections

Globes were postfixed in 4% PFA overnight at 4°C, washed in PBS, and cryopreserved by overnight immersion in 30% sucrose at 4°C. Following embedding in optimal cutting temperature compound (Tissue-Tek, Miles Laboratories), eyes were then frozen on dry ice and sectioned at 13 μm through the dorsal-ventral/superior-inferior axis of the retina onto Superfrost Plus slides (VWR), using a Bright OTF 5000 cryostat (Bright Instruments). Sections were simultaneously blocked and permeabilized by incubation in 5% normal goat serum in PBS with 0.3% Triton X-100 and 2% BSA for 60 min at room temperature. Sections were then incubated in primary antibody (BDNF N-20, rabbit, 1:200, sc-546, Santa Cruz Biotechnology; TrkB, rabbit, 1:300, ab33655, Abcam; RBPMS, guinea pig, 1:500, 1832, PhosphoSolutions; protein kinase Cα, mouse, 1:500, sc-8393, Santa Cruz Biotechnology; vimentin, chicken, 1:500, ab5733, Millipore; calretinin, rabbit, 1:500, ab702, Abcam; calbindin, rabbit, 1:500, ab11426, Abcam; GFP, mouse, 1:500, ab1218, Abcam; GFP, rabbit, 1:500, ab290, Abcam; TUJ1, rabbit, 1:500, ab18207, Abcam) diluted in blocking solution overnight at 4°C, before incubation with secondary antibody (goat anti-mouse AF488, 1:1000, a11029, Invitrogen; goat anti-rabbit AF488, 1:1000, a11034, Invitrogen; goat anti-mouse AF555, 1:1000, a21424, Invitrogen; goat anti-chicken AF568, 1:1000, a11041, Invitrogen; goat anti–guinea pig AF555, 1:1000, a21435, Invitrogen; goat anti-rabbit AF555, 1:1000, a21428, Invitrogen; goat anti-rabbit AF647, 1:1000, a32733, Invitrogen; goat anti-mouse AF647, 1:1000, a21235, Invitrogen) diluted in blocking solution for 120 min at room temperature. Nuclei were counterstained with 4′,6-diamidino-2-phenylindole (DAPI) (1:10,000, D1306, Invitrogen). Sections were imaged using a Leica DM6000 epifluorescence microscope (Leica Microsystems), and high-magnification images were achieved using an SP5 confocal microscope equipped with a 40× lens (Leica Microsystems). RGC counts were measured by counting the mean number of TUJ1-positive cells from three separate 350-μm long sections of retina per animal. Images were adjusted for intensity to facilitate quantification of cell bodies. Original fluorescence intensities are shown.

### I-TASSER molecular modeling of TrkB receptor

I-TASSER is an online platform used to predict protein structure and structure-based function (*63*, *64*). It first identifies structural templates from the Protein Data Bank by multiple threading approach and generates three-dimensional (3D) models for a given sequence. Functional insights can be derived by rethreading the 3D models through the protein function database BioLiP. The conformation of the extended TrkB receptor containing the remaining viral 2A peptide linker was modeled using the online I-TASSER tool (*63*, *64*).

### Statistical analysis

Data were analyzed using Student’s two-paired *t* test or analysis of variance (ANOVA) followed by Bonferroni-modified *t* tests for multiple comparisons; *P* < 0.05 was considered significant. All analyses were performed blind with respect to treatment groups. Sample sizes were calculated using a pilot study and online sample size calculator (https://www.stat.ubc.ca/) adopting a two-sided α level of 0.05 and 80% power.

## SUPPLEMENTARY MATERIALS

Supplementary material for this article is available at http://advances.sciencemag.org/cgi/content/full/7/14/eabd2590/DC1

## Appendix

View/request a protocol for this paper from *Bio-protocol*.
